# Effect of Rosemary (*Rosmarinus officinalis* L.) Supplementation on Probiotic Yoghurt: Physicochemical Properties, Microbial Content, and Sensory Attributes

**DOI:** 10.3390/foods10102393

**Published:** 2021-10-09

**Authors:** Haider I. Ali, Mithun Dey, Azalldeen Kazal Alzubaidi, Sadiq Jaafir Aziz Alneamah, Ammar B. Altemimi, Anubhav Pratap-Singh

**Affiliations:** 1 Department of Food Science, College of Agriculture, University of Basrah, Basrah 61004, Iraq; haider.ali@uobasrah.edu.iq (H.I.A.); ammar.ramddan@uobasrah.edu.iq (A.B.A.); 2 Food Nutrition and Health Program, Faculty of Land & Food Systems, The University of British Columbia, 2205 East Mall, Vancouver, BC V6T 1Z4, Canada; mithun.pharma85@gmail.com; 3 College of Agriculture, University of Misan, Al-Amara 62001, Iraq; ez_aldeen@uomisan.edu.iq; 4 Food Science Department, Agriculture College, Kufa University, Al-Najaf 54001, Iraq; sadiqj.almusawi@uokufa.edu.iq

**Keywords:** antioxidant, total phenolic compound, probiotic, functional food, rosemary, yoghurt

## Abstract

Rosemary (*Rosmarinus officinalis* L.) is a natural aromatic plant that belongs to the family of *Lamiaceae*. The rosemary plant has been utilized to preserve food due to its ability to prevent oxidation and microbial contamination. This study aimed to investigate the effect of fortifying yoghurt with rosemary extracts and probiotic bacteria (LAB) (*Bifidobacterium longum* ATCC15707 and two lactic acid bacteria, *Streptococcus thermophilus* and *Lactobacillus delbrueckii* subsp. *bulgaricus*) on its chemical composition, total phenolic compounds, antioxidant capacity, and sensory properties. The study results revealed significant differences in the total solids, protein, and ash content when rosemary concentration increased beyond 2%. However, there were no significant differences among the treatments in acidity and pH value. The sensory evaluation results indicated that the addition of aqueous extract of rosemary affected the sensory properties of yoghurt (flavour, body and texture, appearance, and overall grade), wherein an increasing concentration of rosemary extract increased score of flavour, body and texture, appearance, and overall grade. On the other hand, rosemary extract did not affect the sensory properties and chemical composition. To sum up, it can be stated that rosemary was used in the preparation of yoghurt with increased health benefits, acceptable sensory attributes, and the production of synbiotic yogurt.

## 1. Introduction

Functional food refers to all kind of foods which deliver important physiological functions and provide nutrients that immensely help in maintaining our body function with additional health advantages [1]. The term ‘functional’ foods has not found any widely accepted definition, because every country has accepted different ideas to define the terminology of functional foods [2]. Functional foods can be defined “as intact foods that include the different categories of foods, for instance, fortified, enhanced, and enriched foods, which when taken daily as part of the various diets at the effective levels produce usefully advantageous effects on health” [1]. Due to the wide propagation of lifestyle-related diseases such as obesity, cancer, diabetes, and cardiovascular diseases such as elevated blood pressure and increased cholesterol, functional foods have generated great attention today [2]. Arguably, all kinds of food items can be considered functional to some extent as they deliver essential energy and nutrients predominantly required for us to maintain a healthy life. However, foods are scientifically termed ‘functional’ only when particular nutrients or active ingredients (like beneficial bacteria) scientifically proven to enhance human health are fortified, enhanced or enriched in intact food products. Functional foods can comprise the fundamentally nutritional elements in natural products such as vegetables and fruits; or can comprise nutrients not naturally found in those products like fortified food products such as vitamin D-fortified milk and vitamin C-fortified fruit juices; probiotics, prebiotics, whole grains, and fibers [3].

The word ‘probiotic’ comes from the Greek term “pro” which means “for” and the Greek term “bios,” which means “life”. Probiotic yoghurt is considered a functional food with a beneficial effect on human health [4]. For example, it can prevent constipation, regarded as one of the most prevalent gastrointestinal problems [5]. The related health advantages include relief of lactose intolerance symptoms, diarrhea treatment, cancer suppression, and reduced blood cholesterol levels. In reality, a product’s matrix can affect the behavior of microbes and thus the microbe’s survival and effects, and thus deserves close attention [6].

Rosemary oil is used as a food seasoning for food [7]. Rosemary oil has traditionally and largely been used as a medicinal herb with number of properties such as anti-inflammatory, analgesic, astringent, antimicrobial, anti-rheumatic, carminative, antifungal, and antioxidant properties [8]. Natural phenolic compounds that have been found in plants and vegetables may decrease the risk of some diseases because of their antioxidant and free-radical inhibition potentials imparted by the benzene ring and the hydroxyl group in their structures [9,10,11]. These phenolic compounds are the result of secondary metabolism in plants, and are often utilized with the intention of therapeutic activity due to their pharmacological features [8,9]. More than 8000 phenolic structures have been described, including essential activities in the propagation and development of the plants, wherein they have a significant function as protection specialists against pathogens, parasites, and provide enhancements to the color of plants [12].

The *Rosmarinus officinalis* L. is familiar as rosemary in Europe and usually known as alecrim in Brazil [13]. This plant’s most recognized pharmacological benefits are identified by its phenolic ingredients: caffeic acid, carnosic acid, rosmarinic acid, and carnosol. As they naturally hold potential antioxidant scavenging effects, carnosol and carnosic acid cover almost 90% of the antioxidant action of rosemary among the phenolic components [13]. Furthermore, rosemary was used to reduce anxiety and depression and measure memory performance during clinical trials of a group of university students [14]. This natural plant is utilized as a flavouring/odour agents in beverages, food items, and cosmetics [15]. *Rosmarinus officinalis* L. extract has been proposed as a possible therapeutic agent for many diseases [16]. Rosemary powder and its lyophilized ethanolic extract at different concentrations were used to formulate fresh cheeses, wherein the plant extract improved phenolics content and antioxidant capacity compared to the control cheese [17]. This study focused on enhancing the functional properties of yoghurt with the addition of different concentrations of rosemary extracts.

## 2. Materials and Methods

Buffalo milk (4.5% protein and 7.0% fat) samples were collected from the local markets and properly stored in the icebox to maintain the temperature at 4 °C. The percent of milk samples’ fat, solids-not-fat (SNF), protein, lactose, density, freezing point, and minerals were determined. The other ingredient, rosemary, was obtained from a medicinal herb garden. One probiotic bacteria *Bifidobacterium longum* ATCC15707 (American Type Culture Collection (ATCC)), two lactic acid bacteria *Lactobacillus delbrueckii* subsp. *bulgaricus*, and *Streptococcus thermophilus* (Sassenage–France) were mixed by 1:1 ratio.

### 2.1. Preparation of Rosemary Aqueous Extract

Rosemary aqueous extract was prepared by the method followed by Abdelfadel and Khalaf [18]. Rosemary sample (25 g) was washed initially using normal tap water, then washed again with distilled water and stored at room temperature, then blended with an electric blender (Vitamix, Black A3300 Ascent Series Smart Blender, Cleveland, OH, USA). Afterward, the rosemary sample was put into a conical flask and extracted with distilled water (100 mL) for 24 h on a vibrator water bath at the three different extraction temperatures of 20 °C, 40 °C, and 60 °C. In the next step aqueous rosemary extract was centrifuged at 3000 rpm for 10 min and the aqueous extract was filtered using Whatman filter paper (GF/A, 110 mm). After that, the extract was filtered of microbes through a Millipore filter extract (0.22 μm micro-filters, Merck & Co., Inc., Darmstadt, Germany) into 10 mL tubes and kept at –20 °C until further usage [19].

### 2.2. Determination of Total Phenolic Content

The total phenolic content (TPC) was estimated by the Folin–Ciocalteau method [20]. We mixed 0.4 mL of the extract in methanol (1 mg/mL) with 2 mL of Folin–Ciocalteau reagent and 1.6 mL of (7%) sodium carbonate. After that, all the samples were shaken gently and placed in a dark place for 90 min. The absorbance of the samples was measured at 750 nm using a spectrophotometer (Shimadzu-UV-160, Shimadzu Research Laboratory Co. Ltd., Tokyo, Japan). Gallic acid monohydrate was used to prepare a standard curve. The TPC was calculated and the result was expressed as mg GAE/g extract.

### 2.3. Determination of Antioxidant Activity

Antioxidant activity of the extracted rosemary samples at the three different temperatures of 20 °C, 40 °C, and 60 °C was estimated depending on free radical scavenging activity using 2, 2-diphenyl-1-picrylhydrazyl (DPPH) radical according to the method suggested by Moo-Huchin [21], where the result is expressed as percentage (%) inhibition.

### 2.4. Minerals Analysis

The rosemary samples extracted at 60 °C were filtered through filter paper (0.45 µm) into a 25 mL volumetric flask and completed to the mark with deionized water. Samples were taken for elemental analysis on the ICP-OES (Model 5100 VDV Agilent) [22].

### 2.5. Determination of Amino Acid Content

The rosemary samples extracted at 60 °C were prepared as per the method of Hwang et al. [23]. Each of the defatted samples (200 mg) were weighed into a glass ampoule and 5 mL of 6N HCl was added. After that, the glass ampoule contents were hydrolyzed in an electric oven preset at 105 °C for 22 h. Oxygen was expelled into the ampoule by passing nitrogen gas into it. Amino acid analysis was undertaken using an amino acid analyzer (Sykam S 433, Shanghai, China). The analysis was carried out with a gas flow rate of 0.5 mL/min at 60 °C, and the reproducibility rate was 3%. The amino acid composition was calculated from the integrated areas of standards peaks and expressed as percentages of the total protein.

### 2.6. Determination of Fatty Acid Composition

Fatty acid (FA) composition was determined for the rosemary samples extracted at 60 °C. Lipid extraction of the samples was performed according to Pratap-Singh et al. [24]. The extract was expressed as crude fat and used for the trans-methylation of the FAs. The FA methyl esters in hexane were then injected into a gas chromatograph (ACME model 610 GC (Young LTN Instrument Co., Anyang, Korea) equipped with a flame ionization detector. The separation of the FA methyl esters was performed using a Famewax™ fused silica capillary column (30 m × 0.25 mm (i.d.), 0.25 μm) (Restek Corporation, Bellefonte, PA, USA). The peak area was measured using a Dani Data Station DDS 1000. Each peak was identified and quantified on the basis of pure methyl ester standards (Restek Corporation, Bellefonte, PA, USA). All analyses were performed in triplicate.

### 2.7. High-Performance Liquid Chromatography (HPLC) Analysis of Phenolic Compounds of Extracts

The phenolic compounds of the extracted rosemary at 60 °C were determined using Agilent 1260 Infinity HPLC series (Agilent, Santa Clara, CA, USA), equipped with a quaternary pump, a Zorbax Eclipse Plus C18 column 100 mm × 4.6 mm i.d., (Agilent Technologies, Santa Clara, CA, USA) operated at 25 °C was used for phenolic compound analysis. The injected volume was 20 µ. VWD detector set at 284 nm. The separation was achieved using a ternary linear elution gradient with (a) HPLC-grade water 0.2% H_3_PO_4_ (*v*/*v*), (b) methanol, and (c) acetonitrile [25].

### 2.8. Preparation of Symbiotic Yoghurt

Symbiotic yoghurt was manufactured by pasteurized buffalo’s milk (15 min/85 °C). then it was cooled down to 42 °C. and then 2 % *w/v* starter culture (*Streptococcus thermophilus* and *Lactobacillus delbrueckii* subsp. *Bulgaricus*) was added at 42 °C and later on, *Bifidobacterium longum* ATCC1570 (2% *w*/*v*) was added at the same temperature. The extracted rosemary at 60 °C was added to the pasteurized buffalo milk at concentrations of 0, 1.5%, 2.0%, 2.5%, and 3.0% (*w*/*v*) according to the study of Ehsani [26], then incubated for 3 h at 42 °C. The yoghurt fortified with different concentration of rosemary extract was stored for (0, 7, and 14 days) at 5 °C. The T1, T2, T3 and T4 were yoghurt fortified with 1.5%, 2.0% 2.5% and 3.0% of *Rosmarinus officinalis* L. aqueous extract, respectively.

### 2.9. Chemical Analysis

Total solids, protein, fat and ash content, and pH value were determined as described by official methods of analysis of AOAC, (2007) [27]. Titratable acidity was evaluated based on Richardson’s study [28].

### 2.10. Microbiological Analysis

The total bacteria count was determined by making a serial dilution to 10 of 1 g of each sample of yogurt. Thereafter, 0.1 mL of each dilution of yoghurt sample was placed on nutrient agar plates and incubated at 35 °C for 48 h. The same procedure was used for counting coliform bacteria and *Staphylococcus aureus*, except that nutrient agar was replaced with MacConkey agar and Mannitol Salt Agar, and all Petri dishes were incubated at 37 °C. Lipolytic bacteria were calculated using Tributyrin Agar (Himedia, Maharashtra, India) and incubated at a temperature of 7 °C for 10 days. The proteolytic bacteria count was enumerated using Caseinate Agar (Himedia, Maharashtra, India) and then incubated at 7 °C for 10 days. *B. longum* were calculated using MRS-NNLP Agar (Himedia, Maharashtra, India) and incubated at a temperature of 37 °C for 72 h in anaerobic conditions. *S. thermophilus* were calculated using M17-Agar (Himedia, Maharashtra, India) and incubated at a temperature of 37 °C for a period of 72 h in aerobic conditions. *Lactobacillus delbruekii* ssp. *Bulgaricus* were enumerated using MRS-Agar (Himedia, Maharashtra, India) and incubated at 37 °C for 72 h in anaerobic conditions. The total number of yeasts and molds was determined according to the method described by Harrigan and MacCane [29].

### 2.11. Sensory Evaluation of Symbiotic Yoghurt

Ten well-trained and experienced panelists participated in the sensory evaluation, in which they evaluated the texture, flavor, appearance and color of the yogurt samples. All the panelists evaluated carefully and scored their responses on a scale of 40 points for texture and 40 points for flavor, 10 points for appearance and 10 points for color [30].

### 2.12. Statistical Analysis

All the experimental data obtained (means of three replicates) were statistically analyzed by one-way analysis of variance (ANOVA test) using SPSS^®^ 13.0 (Statistical Package for the Social Sciences) (2005). Statistical differences were considered significant at (*p* ≤ 0.05).

## 3. Results and Discussion

### 3.1. Effect of Extraction Temperature on the Phenolic Content and Antioxidant Activity of Rosemary Extract

**Figure 1:**
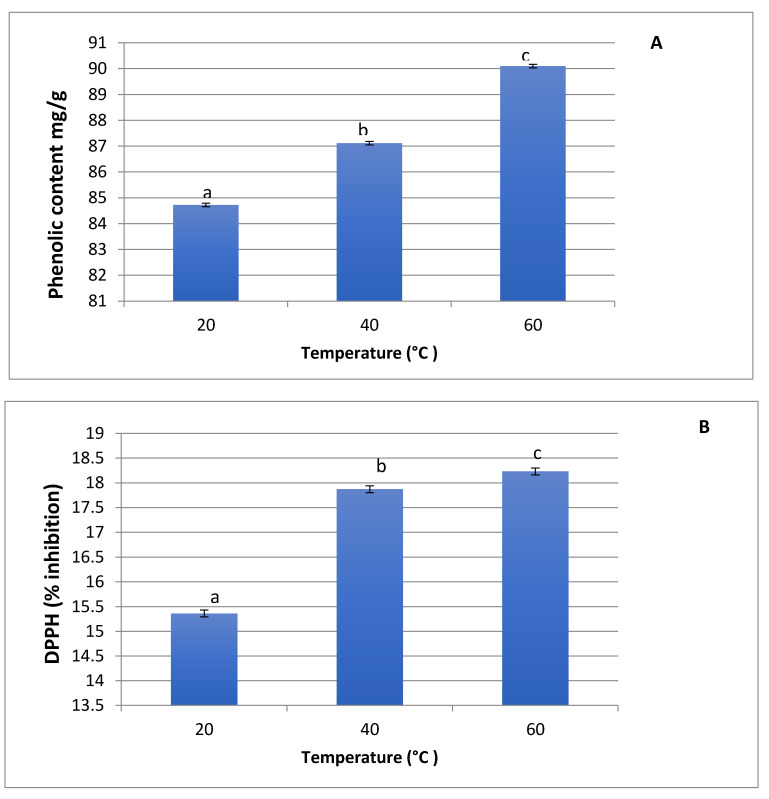
(**A**) The total phenolic content and (**B**) antioxidant activity of rosemary plant extracts (a–c denotes means with same letters are not statistically significant *p* > 0.05).

The phenolic content of rosemary plant extracts increased from 85 to 90 mg GAE/g as the extraction temperature increased from 20 °C to 60 °C, respectively (Figure 1a). There was a statistically significant difference (*p* < 0.05) between the amount of total phenolic content extracted from the rosemary plant at different temperatures. The corresponding antioxidant activity of rosemary plant extracts also increased from 15% to 18% as extraction temperatures increased from 20 °C to 60 °C, respectively (Figure 1b).

Obtaining a higher quantity of phenolic compounds during high temperatures indicated the ability of temperatures and their efficiency to extract the multiple phenolic compounds. According to Erkan [31], when comparing rosemary extract and blackseed essential oil, the rosemary extract was found to have a higher phenolic content than blackseed essential oil in all three test methods applied in their experiment. Gomaa [19] found that aqueous extract of rosemary plant showed higher phenolic content (104.99 mg GAE/g extract) than other plant extracts studied including dill, garlic, flaxseeds, and oat were extracted by water solvent. Furthermore, the total phenolic compound of rosemary plant extract extracted by ethanolic (10% dimethylsulfoxide; DMSO) extract exhibited a lower value (46.89 mg GAE/g extract) than aqueous extracted rosemary extract. Our study results provided evidence that rosemary aqueous extract contained a substantial amount of total phenolic compounds, agreeing with another prior study of Khalil and Gomaa [32].

Our results confirm the utilization of 60 °C as the extraction temperatures for further parts of this study. One may argue that a higher temperature than 60 °C may be utilized, however, a higher temperature than 60 °C is generally not preferred as the high temperature adversely affect the phenolic and antioxidant content [33] more than their ability to increase extraction efficiency. Trojakova’s study [34] indicated that the rosemary extract showed higher antioxidant activity under the test conditions at 40 °C or 60 °C than the temperature at 100 °C. Another research work [35] revealed that the extraction of polyphenolic compounds from rosemary extract was the most efficient at the extraction temperature of 60 °C compared to 70 °C and 90 °C. This confirms that moderate temperature (60 °C) was enough to extract phenolic compounds that have antioxidant activity and thus give a significantly higher efficacy.

### 3.2. Characterization of the Rosemary Aqueous Extract Extracted at 60 °C

In terms of mineral composition, the rosemary extract contained different macro-elements such as sodium, potassium, calcium, phosphorus, magnesium, and micro-elements for instance, iron, copper, manganese, zinc etc. Among the identified macro-elements, the content of potassium was the highest and the lowest amount was observed in magnesium. For micro-elements, the highest quantity was reported for Iron, and copper content was the least (Table 1). The study result was almost similar to the Boix et al. [35] where the growth and development of *Rosmarinus officinalis* plants occurred under the specified conditions and Daghestani et al. [36]. The rosemary extract contained both the essential amino acids (EAAs) and non-essential amino acids (NEAAs), including histidine, isoleucine, leucine, lysine, Table 1 shows the percentage of amino acids in the rosemary plant extract, as the extract contained the amino acids Histidine, Isoleucine, Leucine, and Liysine.

Methionine, phenylalanine, threonine, tyrosine, valine, alanine, and arginine. The quantity of phenylalanine (11.61) was the maximum and the methionine (1.81) had the minimum volume among all the essential acids. The study results were similar to those obtained by Peixoto et al. [37]. Respectively, the results obtained were similar to those obtained.

The rosemary extract also contained fatty acids comprising myristic acid, palmitic acid, palmitoleic, stearic acid, oleic acid, linoleic acid, Linolenic acid, arachidic acid, gondoic acid, and behenic acid,. This study result also exhibited that the amount of unsaturated fatty acid (75.54 g) was higher than the saturated fatty acid (24.65 g) and the results were similar to the result found in the Popescu experiment [38].

**Figure 2:**
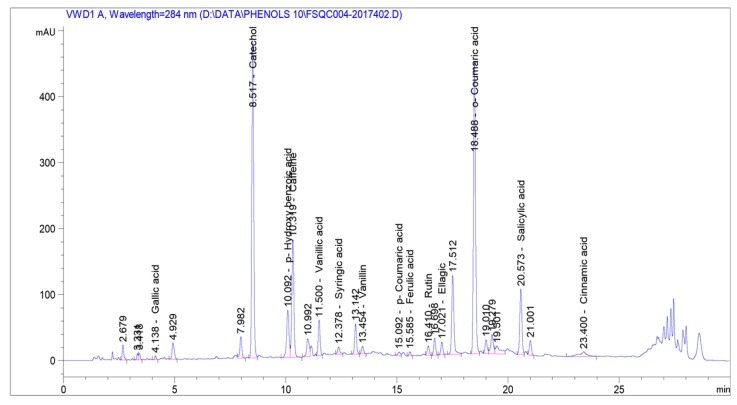
High-performance liquid chromatography (HPLC) chromatogram of phenolic compounds in *Rosmarinus officinalis.* L. (detection at 284 nm). *X*-axis = retention time, min = minutes; and *Y*-axis = detector signal.

Table 1 describes the chemical composition (phenolic compounds, minerals, amino acids and fatty acids) of the rosemary aqueous extract at 60 °C temperature. We quantified 14 different phenolic compounds in the rosemary plant extract. Figure 2 shows the chromatogram of phenolic compounds in *Rosmarinus officinalis* L. where 14 phenolic compounds were detected at the wavelength of 284 nm which included gallic acid (4.1 min), catechol (8.5 min), p-hydroxybenzoic acid (10.0 min), caffeine (10.3 min), vanillic acid (11.5 min), syringic acid (12.3 min), vanillin (13.4 min), p-coumaric acid (15.0 min), ferulic acid (15.5 min), rutin (16.4 min), ellagic (17.0 min), o-coumaric acid (18.4 min), salicylic acid (20.5 min), and cinnamic acid (23.4 min). Amongst all the detected compounds, the quantity of catechol was the highest (1474.25 mg/g) and the lowest amount (3.18 mg/g) was p-coumaric acid, while caffeic acid, and benzoic acid were not detected. Other compounds in major concentration (above 200 mg/g) identified were p-hydroxy benzoic acid, caffeine, o-coumaric acid, and salicylic acid. A previous study [30] identified 11 different compounds (gallic acid, caffeic acid, ferulic acid, rosmarinic acid, coumaric acid, carnosol, carnosic acid, hesperidin, luteolin, apigenin and genkwanin) in extracts of rosemary plants. Kostikova [39] found 22 phenolic compounds in aqueous ethanolic extracts from *Sorbaria pallasii* leaves using HPLC analysis. Nour et al. [40] developed an efficient and precise HPLC method to quantify pharmacologically active phenolic compounds such as vanillic, gallic acid, syringic, p-coumaric, ferulic, ellagic, and salcyilic acid in walnut leaves, where the content of ellagic acid was much higher than other phenolic acids. The study result determined the phenolic compounds were similar to the phenolic acids found in our experiment and rosemary extract is capable of showing extensive antioxidant activity like walnut leaves. A study by Gini and Jeya [41] showed that the identification of a number of phenolic compounds, for instance, gallic acid, catechol, and vanillin occurred in the active fractions of ethyl acetate extract of *Salvinia molesta* and the identified phenolic compounds were found to depict antioxidant activity that was able to reduce or inhibit oxidative damage resulting from free radicals. The outcome of this research study has similar effects to our present study.

### 3.3. Characterization of the Yoghurt Fortified with Rosemary Aqueous Extract

The chemical composition of yoghurt prepared with different concentrations of rosemary aqueous extract is illustrated in Table 2. The addition of 1.5%, 2.0%, 2.5%, and 3.0% aqueous extract of rosemary markedly increased the fat, protein, ash content, and increased pH value of yoghurt of the treatment groups (T1, T2, T3, and T4) compared with the control group during storage time, whereas the moisture content of the control group was higher than all treatment groups in days 0, 7, and 14. Among all the treatment groups, it is noticeable that the moisture content of the samples decreased gradually when the concentrations of rosemary aqueous extract increased proportionally on 0, 7 days and also after 14 days of storage. This might be caused by evaporation during storage where the rosemary aqueous extracts probably are not effected by the controlling of moisture of the samples.

The fat content of treatment groups showed a gradual rise with the increase of their concentrations compared to the control group and the highest percentages of fat content observed in all the treatment groups including the control group after 14 days of preservation. There were no statistically significant differences (*p* > 0.05) between rosemary treatment groups and the control group.

The protein content of yoghurt treatments which include T1, T2, T3, and T4 were 4.41%, 4.45%, 4.51%, and 4.57%, respectively, in 0 days, and the percentages of protein content were attained maximal after 14 days of storage compared to 0 days and 7 days storage. The explanation for this rise can be attributed to the continued decrease in the moisture content of the yoghurt during storage times. There was no statistically significant difference (*p* > 0.05) in this study among the control and treatment groups.

Likewise with the protein content, there was an increase exhibited in the ash content of all yoghurt treatments after finishing 14 days storage timeframe without statistically significant differences (*p* > 0.05). After 14 days preservation, the ash content were also reached maximum in all the treatment groups including control group. The ash content of the samples was increased moderately when the concentrations of rosemary aqueous extract increased proportionally. Ash content has been affected by the addition of rosemary aqueous extracts in the yoghurt samples compared to the control one.

The pH values were low for all the treatment groups and control groups after 14 days of storage; however, the treatment groups’ pH value was higher than the control group. Joung [42] also found that the pH values of all yoghurt treatments were decreased during the 14 days of storage and the pH reduction during storage also could be the cause of lactose conversion into lactic acid in storage time [43]. The rosemary aqueous extracts mixed treatment groups restricted the bacterial growth of yoghurt and subsequently kept pH higher than the pH of a control group.

The titratable acidity of all the treatments and control samples were determined after storing at a temperature of 4 °C for a period of 0, 7, and 14 days (Table 2). There was no statistically significant difference in the titration acidity of all the studied samples with the progress of the storage periods. In the titration acidity study, it was noticed that the titration acidity values of the control samples were higher compared to all treatment groups of samples. This may be the reason why the different treatment groups containing different concentrations of rosemary extracts caused partial or complete inactivation of some microorganisms, especially for Gram-positive bacteria by absorbing rosemary extracts that affect the bacterial cell surface. Gorissen et al. [44] found that the permeability of the cytoplasm membrane to bacteria was increased at 4 °C temperature with the presence of extracts. Another study by Smith-Palmer [45] also indicated the ability of the plant extracts in the produced yogurt to form a protective layer (coat around bacterium) against micro-organisms, as it caused an obstruction to the transfer of the active compounds in the plant extracts added to the active or inactive sites in the bacteria due to the low and restricted water content of the product. Moreover, Xu [46] state that the refrigerator temperature does not affect the growth of lactic acid bacteria.

Table 3 represents the total number of bacteria, coliform bacteria, *Staphylococcus aureus*, lipolytic bacteria, proteolytic bacteria, molds, and yeasts in the stored yogurt samples at 5 °C for 0, 7, and 14 days storage. Total bacterial count increased from 6.18 CFU/gm yogurt in 0 days to 8.16 CFU/g yoghurt after 14 days for control samples where all the treatment group of samples showed the lower total bacteria count compared to the control on the 14th day. The control samples showed a higher coliform content compared to other yogurt treatments. As the storage days progressed, it was clearly observed that the control group had the highest bacterial concentration and with an increase in the concentration of rosemary extract, the coliform bacterium content was gradually decreased. In terms of *Staphylococcus aureus,* there was no bacterial production in the treatment groups after both 0 and 7 days however, the production of *Staphylococcus aureus* reached up to 3.07 CFU/gm after 7 days storage in the control samples. However, the content of *Staphylococcus aureus* was higher (3.43 CFU/gm) in the control group compared to all the rosemary mixed treatment groups. Considering the preparation of lipolytic bacteria and proteolytic bacteria, there were no noticeable differences between the different transactions and storage periods. In 0 days, no lipolytic and proteolytic bacteria were formed in the control samples; however, the numbers of bacteria reached the maximum level at the 14 days. For rosemary treatment groups, the bacteria numbers were decreased markedly after 14 days of storage because of the effects of rosemary aqueous extract. The rosemary extract also positively affected the total numbers of yeasts and molds, as the storage periods of 0 and 7 days did not provide any fungal growth for all the studied samples. After 7 days, the control samples showed fungal growth and the yeast and mold numbers reached 2.32 CFU/gm at the 14 days, while the treatment groups experienced a low level of fungal growth and 1.30 CFU/gm was the lowest for T4 among all the treatments. On the other hand, the rosemary extract also had a negative effect on the total number of probiotic bacteria (*Bifidobacterium longum*) and the starter culture (*L. bulgaricus* and *S. thermophilus*). The probiotic strain and the starter culture did not show the same stability during storage.

### 3.4. Sensory Evaluation of Yoghurt Fortified with Rosemary Aqueous Extract

Results of sensory evaluation properties of yoghurt revealed that the adding of rosemary as an aqueous extract affected the flavour, body and texture, appearance, and overall acceptance of yoghurt samples (Table 4). In addition, all yoghurt fortified with different concentrations of rosemary aqueous extract had acceptable flavour, body, texture, and appearance scores except the treatment group T4 (for which flavour score was unacceptable). Furthermore, the rosemary extract was used to fortify yoghurt to enhance its nutritional and biological values without any noticeable adverse effect on its acceptability. Ayad et al. [30] observed that the different concentrations of aqueous rosemary extract provided increased health benefits and were acceptable with varying effects on sensory attributes. All yoghurt treatment’s sensory evaluation scores were reduced unevenly during the storage period, which might be attributed to lactic acid production and aromatic compounds such as acetone, acetaldehyde, and diacetyl [47]. The amalgamation of probiotic, prebiotic, and aqueous rosemary extract positively affected synbiotic yoghurt treatments’ sensory scores. This agrees with the findings of previous researchers that a desirable effect on sensory properties will be gained by using probiotic species in dairy products [48].

## 4. Conclusions

This study revealed that the fortification of yoghurt with the treatment groups T2 and T3 of rosemary aqueous extract showed slightly increased protein, ash, and total solid content; however, they exhibited potential antioxidant activity and held a substantial amount of total phenolic content as well. Furthermore, there was no change in the yogurt’s color, taste, and overall acceptability scores for the treatment group T1, whereas the treatment group T4 showed the lowest overall acceptance among all treatments. There was a good correlation between the total phenolic content and the antioxidant activity, which supports the concept of phenols as contributors to the antioxidant activity of the aqueous extract of rosemary. Previously, scientists conducted very few related studies about adding aqueous rosemary extract in probiotic fermented milk and yogurt to improve its nutrient quality and functional properties. Hence, this study examined the properties of rosemary extracts and suggested the use of an aqueous extract of rosemary as fortified fermented milk via a suitable, cost-effective approach for making products that improve public health.

## Figures and Tables

**Table 1:** (**a**) Phenolic composition, (**b**) mineral (elemental) composition, (**c**) amino-acid profile and (**d**) fatty acid profile of *Rosmarinus officinalis* L. extract.

(a) Phenolic Compounds	Conc.(mg/100 g) ^1^
Gallic acid	4.31 ± 0.480
Catechol	1474.25 ± 5.833
P-Hydroxy benzoic acid	309.65 ± 1.661
Caffeine	235.66 ± 2.361
Vanillic acid	64.38 ± 1.103
Caffeic acid	ND ^2^
Syringic acid	13.00 ± 0.636
Vanillin	9.38 ± 0.791
p- Coumaric acid	3.18 ± 0.643
Ferulic acid	4.91 ± 0.127
Rutin	54.25 ± 0.579
Ellagic acid	42.49 ± 0.360
Benzoic acid	ND ^2^
O-Coumaric acid	232.31 ± 1.124
Salicylic acid	271.54 ± 1.025
Cinnamic acid	14.26 ± 0.678
**(b)** **Minerals**	**Conc.(mg/100 g) ^1^**
Macro-elements	
Sodium (Na)	92.31 ± 0.577
Potassium (K)	2035.51 ±3.89
Calcium (Ca)	1246.35 ± 4.49
Phosphorus (P)	477.29 ± 5.51
Magnesium (Mg)	45.95 ± 1.05
Micro-elements	
Iron (Fe)	45.36 ± 1.09
Copper (Cu)	0.59 ± 0.05
Manganese (Mn)	4.59 ± 0.88
Zinc (Zn)	7.42 ± 0.98
**(c)** **Amino acids**	**Conc.(mg/100 g) ^1^**
Essential Amino Acids (EAAs)	
Histidine	4.19 ±0.43
Isoleucine	2.92 ± 0.29
Leucine	5.82 ± 0.53
Lysine	5.79 ± 0.12
Methionine	1.81 ± 0.09
Phenylalanine	11.61 ± 0.36
Threonine	2.62 ± 0.09
Tyrosine	4.92 ± 0.32
Valine	4.82 ± 0.29
Non-essential Amino Acids (NEAAs)	
Alanine	5.88± 0.63
Arginine	4.04 ± 0.11
**(d)** **Fatty acids**	**Conc.(mg/100 g) ^1^**
Myristic acid (C14:0)	3.48 ± 0.36
Palmitic acid (C16:0)	15.36 ± 0.91
Palmitolic (C16:1)	3.79 ± 0.38
Stearic acid (C18:0)	4.36 ± 0.44
Oleic acid (C18_:1_)	41.67 ± 1.12
Linoleic acid (C18:2)	7.57 ± 0.85
Linolenic acid (C18:3)	18.87 ± 1.09
Arachidic acid (C20:0)	0.84 ± 0.07
Gondoic acid (C20:1)	4.48 ± 0.54
Behenic acid (C22:0)	0.84 ± 0.09
SFA ^3^	24.65 ± 0.27
USFA ^4^	75.54 ± 1.08
USFA ^4^/AFA ^3^	3.23 ± 0.98

^1^ Values are expressed in mg/100 g extract as mean ± standard deviation. ^2^ ND: Not Detected. ^3^ SFA: Saturated fatty acid; ^4^ USFA: Unsaturated fatty acid.

**Table 2:** Chemical composition, pH, and acidity of yoghurt fortified with different concentration of rosemary extract (mean ± standard deviation) during storage (0, 7, and 14 days).

Treatment	Storage (Days)	Control	T1	T2	T3	T4
Moisture (%)	0	89.42 ± 0.07 ^d^	88.55 ± 0.07 ^cd^	87.67 ± 0.07 ^bc^	86.83 ± 0.07 ^ab^	85.52 ± 0.07 ^a^
	7	89.13 ± 0.07 ^d^	88.24 ± 0.07 ^cd^	87.48 ± 0.07 ^cd^	86.63 ± 0.07 ^ab^	85.26 ± 0.07 ^a^
	14	88.27 ± 0.07 ^d^	87.85 ± 0.07 ^cd^	86.77 ± 0.07 ^cd^	85.80 ± 0.07 ^ab^	84.72 ± 0.07 ^a^
Fat (%)	0	6.49 ± 0.07 ^d^	6.54 ± 0.07 ^cd^	6.60 ± 0.07 ^bc^	6.70 ± 0.07 ^ab^	6.77 ± 0.07 ^a^
	7	6.52 ± 0.07 ^d^	6.56 ± 0.07 ^cd^	6.65 ± 0.07 ^cd^	6.73 ± 0.07 ^ab^	6.79 ± 0.07 ^a^
	14	6.53 ± 0.07 ^d^	6.57 ± 0.07 ^cd^	6.67 ± 0.07 ^cd^	6.75 ± 0.07 ^ab^	6.82 ± 0.07 ^a^
Protein (%)	0	4.35 ± 0.03 ^d^	4.41 ± 0.03 ^cd^	4.45 ± 0.03 ^bc^	4.51 ± 0.03 ^ab^	4.57 ± 0.03 ^a^
	7	4.37 ± 0.03 ^d^	4.42 ± 0.03 ^cd^	4.48 ± 0.03 ^bc^	4.53 ± 0.03 ^ab^	4.59 ± 0.03 ^a^
	14	4.39 ± 0.03 ^d^	4.44 ± 0.03 ^cd^	4.50 ± 0.03 ^bc^	4.56 ± 0.03 ^ab^	4.61 ± 0.03 ^a^
Ash (%)	0	0.60 ± 0.04 ^d^	0.64 ± 0.04 ^cd^	0.69 ± 0.04 ^bc^	0.73 ± 0.04 ^ab^	0.79 ± 0.04 ^a^
	7	0.62 ± 0.04 ^d^	0.65 ± 0.04 ^cd^	0.71 ± 0.04 ^bc^	0.75 ± 0.04 ^ab^	0.81 ± 0.04 ^a^
	14	0.63 ± 0.04 ^d^	0.67 ± 0.04 ^cd^	0.72 ± 0.04 ^bc^	0.77 ± 0.04 ^ab^	0.82 ± 0.04 ^a^
pH value	0	4.62 ± 0.02 ^a^	4.63 ± 0.02 ^a^	4.65 ± 0.02 ^a^	4.70 ± 0.02 ^a^	4.75 ± 0.02 ^a^
	7	4.58 ± 0.02 ^a^	4.60 ± 0.02 ^a^	4.62 ± 0.02 ^a^	4.66 ± 0.02 ^a^	4.70 ± 0.02 ^a^
	14	4.56 ± 0.02 ^a^	4.59 ± 0.02 ^a^	4.60 ± 0.02 ^a^	4.62 ± 0.02 ^a^	4.68 ± 0.02 ^a^
Titration Acidity	0	0.9 ± 0.01 ^a^	0.89 ± 0.01 ^a^	0.86 ± 0.01 ^a^	0.81 ± 0.01 ^a^	0.75 ± 0.01 ^a^
	7	0.92 ± 0.01 ^a^	0.91 ± 0.01 ^a^	0.9 ± 0.01 ^a^	0.86 ± 0.01 ^a^	0.81 ± 0.01 ^a^
	14	0.94 ± 0.01 ^a^	0.92 ± 0.01 ^a^	0.91 ± 0.01 ^a^	0.89 ± 0.01 ^a^	0.83 ± 0.01 ^a^

T1: yoghurt fortified with 1.5% of *Rosmarinus officinalis* L. aqueous extract. T2: yoghurt fortified with 2.0% of *Rosmarinus officinalis* L. aqueous extract. T3: yoghurt fortified with 2.5% of *Rosmarinus officinalis* L. aqueous extract. T4: yoghurt fortified with 3.0% of *Rosmarinus officinalis* L. aqueous extract. ^a–d^ Means with same superscripts across a row are not significantly different *p* > 0.05.

**Table 3:** Log of the total bacterial, coliform, *Staphylococcus aureus*, lipolytic, proteolytic, fungi, and molds in yogurt (mean ± standard deviation).

Sample	Microbial Content Bacterial Species	Bacterial Count over the Storage Period (Day)		
		0 Day	7 Day	14 Day
Control	Total count bacteria	6.18 ± 0.06 ^a,x^	7.12 ± 0.02 ^b,x^	8.16 ± 0.19 ^c,x^
	Coliform	-	2.39 ± 0.07 ^a,x^	3.96 ± 0.16 ^b,x^
	*S. aureus*	-	3.07 ± 0.07 ^b,x^	3.43 ± 0.01 ^a,x^
	Lipolytic bacteria	-	2.04 ± 0.01 ^a,x^	3.43 ± 0.01 ^b,x^
	Proteolytic bacteria	-	2.14 ± 0.03 ^a,x^	3.33 ± 0.03 ^b,x^
	Yeasts and molds	-	1.69 ± 0.03 ^a,x^	2.32 ± 0.04 ^b,x^
	*L. bulgaricus*	6.40 ± 0.01 ^b,x^	5.90 ± 0.03 ^b,x^	5.44 ± 0.02 ^a,x^
	*S.thermophilus*	6.12 ± 0.02 ^b,x^	6.12 ± 0.02 ^b,x^	5.24 ± 0.02 ^a,x^
	*B. longum*	8.90 ± 0.02 ^b,x^	8.71 ± 0.02 ^b,x^	8.18 ± 0.03 ^a,x^
T1	Total count bacteria	6.18 ± 0.06 ^a,x^	6.55 ± 0.04 ^b,y^	6.69 ± 0.06 ^b,y^
	Coliform	-	2.26 ± 0.01 ^a,y^	2.56 ± 0.05 ^b,y^
	*S. aureus*	-	1.34 ± 0.05 ^a,y^	1.95 ± 0.04 ^b,y^
	Lipolytic bacteria	-	2.04 ± 0.01 ^a,x^	2.21 ± 0.01 ^a,y^
	Proteolytic bacteria	-	1.48 ± 0.01 ^a,y^	1.91 ± 0.06 ^b,y^
	Yeasts and molds …	-	1.07 ± 0.02 ^a,y^	1.32 ± 0.02 ^b,y^
	*L. bulgaricus*	6.14 ± 0.02 ^c,x^	5.84 ± 0.04 ^b,y^	5.35 ± 0.02 ^a,y^
	*S. thermophilus*	6.04 ± 0.03 ^c,x^	5.60 ± 0.01 ^b,y^	5.22 ± 0.02 ^a,y^
	*B. longum*	8.93 ± 0.01 ^c,x^	8.46 ± 0.01 ^b,y^	7.98 ± 0.03 ^a,y^
T2	Total count bacteria	6.18 ± 0.05 ^a,x^	6.47 ± 0.03 ^b,y^	6.61 ± 0.01 ^c,y^
	Coliform	-	2.23 ± 0.09 ^a,y^	2.54 ± 0.02 ^b,y^
	*S. Aureus*	-	1.24 ± 0.04 ^a^	2.27 ± 0.02 ^b,z^
	Lipolytic bacteria	-	2.04 ± 0.02 ^a^	1.69 ± 0.04 ^b,z^
	Proteolytic bacteria	-	1.32 ± 0.04 ^a^	1.94 ± 0.03 ^b,y^
	Yeasts and molds …	-	1.03 ± 0.02 ^a^	1.31 ± 0.01 ^b,y^
	*L. bulgaricus*	6.13 ± 0.02 ^c,x^	5.76 ± 0.03 ^b^	5.31 ± 0.02 ^a,y^
	*S. thermophilus*	6.04 ± 0.03 ^c,x^	5.58 ± 0.01 ^b^	5.12 ± 0.02 ^a,z^
	*B. longum*	8.91 ± 0.02 ^c,x^	8.36 ± 0.03 ^b^	7.83 ± 0.02 ^a,y,z^
T3	Total count bacteria	6.18 ± 0.05 ^a,x^	6.43 ± 0.02 ^a,y^	6.58 ± 0.03 ^a,y^
	Coliform	-	2.13 ± 0.02 ^a,z^	2.52 ± 0.03 ^a,y^
	*S. Aureus*	-	1.24 ± 0.04 ^a^	2.23 ± 0.01 ^b,z^
	Lipolytic bacteria	-	2.05 ± 0.02 ^a^	1.62 ± 0.03 ^b,z^
	Proteolytic bacteria	-	1.36 ± 0.04 ^a^	1.94 ± 0.04 ^b,y^
	Yeasts and molds …	-	1.01 ± 0.02 ^a^	1.33 ± 0.02 ^b,y^
	*L. bulgaricus*	6.12 ± 0.01 ^c,x^	5.71 ± 0.03 ^b^	5.31 ± 0.02 ^a,y^
	*S. thermophilus*	6.03 ± 0.03 ^c,x^	5.57 ± 0.01 ^b^	5.11 ± 0.02 ^a,z^
	*B. longum*	8.86 ± 0.01 ^c,x^	8.32 ± 0.01 ^b^	7.74 ± 0.01 ^a,z^
T4	Total count bacteria	6.18 ± 0.045 ^a,x^	6.33 ± 0.03 ^a,z^	6.43 ± 0.02 ^a,z^
	Coliform	-	2.21 ± 0.01 ^a,y^	2.48 ± 0.04 ^b,z^
	*S. Aureus*	-	1.17 ± 0.04 ^a^	2.21 ± 0.01 ^b.z^
	Lipolytic bacteria	-	2.01 ± 0.03 ^a^	1.60 ± 0.02 ^b,z^
	Proteolytic bacteria	-	1.26 ± 0.04 ^a^	1.84 ± 0.01 ^b,z^
	Yeasts and molds …	-	1.00 ± 0.07 ^a^	1.30 ± 0.03 ^b,y^
	*L. bulgaricus*	6.12 ± 0.01 ^c,x^	5.71 ± 0.03 ^b^	5.31 ± 0.02 ^a,y^
	*S. thermophilus*	6.04 ± 0.03 ^c,x^	5.53 ± 0.06 ^b^	5.04 ± 0.03 ^a,z^
	*B. longum*	8.90 ± 0.01 ^c,x^	8.29 ± 0.02 ^b^	7.62 ± 0.02 ^a,z^

T1: yoghurt fortified with 1.5% of Rosmarinus officinalis L. aqueous extract. T2: yoghurt fortified with 2.0% of Rosmarinus officinalis L. aqueous extract. T3: yoghurt fortified with 2.5% of Rosmarinus officinalis L. aqueous extract. T4: yoghurt fortified with 3.0% of Rosmarinus officinalis L. aqueous extract. ^a–c^ Means with same superscripts across a row are not significantly different. ^x–z^ Means with same superscripts across a microbial type between different treatments are not significantly different *p* > 0.05.

**Table 4:** Sensory evaluation of symbiotic yoghurt fermented milk.

Properties	Storage (Day)	Control	T1	T2	T3	T4
Flavor (40)	0	36	35	35	34	34
	7	34	33	32	32	31
	14	33	31	30	29	29
Average		34.3 ± 1.527 ^a^	33 ± 2.08 ^a^	32.3 ± 2.517 ^a^	31.6 ± 2.516 ^b^	31.3 ± 2.516 ^b^
Texture (40)	0	36	35	35	34	34
	7	33	32	31	31	30
	14	32	30	30	30	29
Average		33.6 ± 2.081 ^a^	32.3 ± 2.516 ^a^	32 ± 2.645 ^a^	31.6 ± 2.081 ^b^	31 ± 2.645 ^b^
Color (10)	0	7	6	5	4	4
	7	6	4	3	2	1
	14	6	3	2	2	1
Average		6.3 ± 0.577 ^a^	4.3 ± 1.527 ^a^	3.3 ± 1.527 ^b^	2.6 ± 1.15 ^b^	2 ± 1.73 ^c^
Appearance (10)	0	6	5	5	4	4
	7	5	3	2	2	1
	14	3	2	1	1	1
Average		4.6 ± 1.527 ^a^	3.3 ± 1.527 ^a^	2.6 ± 2.081 ^b^	2.3 ± 1.527 ^b^	2 ± 1.732 ^c^

^a–c^ Means with same superscripts across a row are not significantly different.

## Data Availability

All data are reported in this manuscript.
